# An uncommon case of a rapidly growing intracardiac mass

**DOI:** 10.21542/gcsp.2025.66

**Published:** 2025-12-31

**Authors:** Afendoulis Dimitrios, Matthaios Didaggelos, Perreas Konstantinos, Kartalis Athanasios

**Affiliations:** 1 Cardiology Department, General Hospital of Chios “Skylitseion”, Chios, Greece; 2 Onassis Cardiac Surgery Center, Athens, Greece; 3 AHEPA University Hospital of Thessaloniki, Cardiology Department

## Abstract

Cardiac myxomas typically demonstrate slow growth rates, with rapid enlargement raising suspicion for malignancy. We present a 77-year-old male with a history of non-Hodgkin lymphoma who developed symptomatic atrial fibrillation. Transthoracic echocardiography revealed a left atrial mass measuring 2.5 cm that was absent six months earlier, suggesting a growth rate of approximately 5 mm/month. Multimodality imaging including transesophageal echocardiography, cardiac CT, and MRI showed features suggestive of myxoma but could not definitively exclude lymphoma recurrence given the patient’s oncological history and atypical growth pattern. Thoracoscopic resection was successfully performed. Histopathology confirmed a cardiac myxoma with central necrosis—a rare finding occurring in less than 3% of cases—which likely contributed to the unusually rapid growth. This case highlights the diagnostic value of multimodality imaging and the importance of multidisciplinary decision-making in managing complex cardiac masses.

## Case presentation

A 77-year-old male presented to our cardiology clinic with dyspnea and palpitations secondary to recurrent atrial fibrillation. His medical history included hypertension managed with amlodipine and olmesartan, and coronary artery disease with a non-STEMI seven years prior, for which he underwent drug-eluting stent placement to the right coronary artery and was maintained on aspirin and statin therapy.

**Figure 1.**
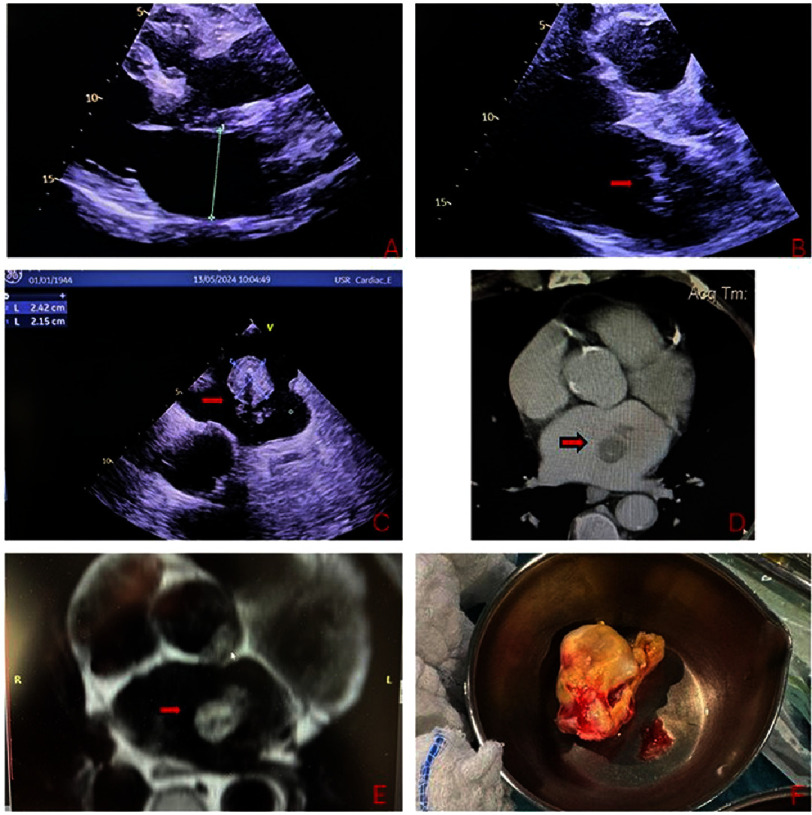
A: Transthoracic heart echo, long axis view depicting the absence of cardiac mass during the first evaluation of the patient (6 months ago). **1B:** Transthoracic heart echo, long axis view depicting the presence of the cardiac mass (red arrow) during the present echo study of the patient. **1C:** Transesophageal heart ultrasound depicting the presence of the intracardiac mass (red arrow), dimensions 2,15×2,4 cm. **1D:** Cardiac computed tomography angiography depicting the cardiac mass within the left atrium (red arrow). **1E**: Cardiac Magnetic resonance depicting the presence of the cardiac mass within the left atrium with low signal on both T1, T2 sequences (red arrow). **1F**: Cardiac mass appearance after its surgical removal.

Additionally, the patient had a history of neck non-Hodgkin lymphoma treated with chemotherapy 15 years prior, with ongoing hematology follow-up. Following unsuccessful attempts at rhythm control with amiodarone, a rate control strategy using an oral beta-blocker was initiated. However, adequate rate control was not achieved, and anticoagulation was transitioned from aspirin to apixaban.

Transthoracic echocardiography (Figure 1) revealed an intracardiac mass that had not been present on imaging performed six months earlier at our institution. Subsequent transesophageal echocardiography demonstrated a mass measuring 2.45 × 2.15 cm with limited mobility, attached to the mid-portion of the posterior left atrial wall. There were no obstructive features. Additional findings included mild mitral regurgitation and a left atrial appendage free of thrombus.

Laboratory testing revealed no significant abnormalities apart from mild anemia. Contrast-enhanced computed tomography of the brain, chest, and abdomen confirmed a heterogeneous spherical mass with irregular borders within the left atrium, measuring approximately 2.5 cm in diameter and attached to the posterior wall. Internal calcifications were present. No extracardiac lesions or nodules were identified.

Cardiac MRI demonstrated low signal intensity on T1 and T2 sequences with contrast enhancement and evidence of hemorrhage. The rapid appearance of a cardiac mass in a patient with a history of malignancy raised significant concern. Following multidisciplinary discussion within the Heart Team and consultation with the patient, thoracoscopic tumor resection was recommended. The operation was performed successfully, and the patient was discharged on postoperative day five following an uneventful recovery.

Histopathological examination revealed a heterogeneous mass with a maximal diameter of four cm, featuring a fibrous capsule, foci of osteoid formation, and necrotic material with calcified and hemorrhagic regions—likely contributing factors to the rapid size increase. The mass was identified as a myxoma, particularly evident in the peripheral regions, with a central necrotic core and calcified and hemorrhagic territories. No malignant cells were identified. At six-week follow-up, the patient remained asymptomatic in sinus rhythm on beta-blocker therapy.

## Discussion

Cardiac myxoma (CM) remains the second most common benign primary cardiac tumor, occurring most frequently in the left atrium. CM may present sporadically or as part of familial syndromes, with a mean age at diagnosis of 50 years old^1,2^.

Symptoms include chest pain, palpitations and dyspnea, obstructive phenomena or regurgitation of the mitral valve, heart failure, central nervous system or peripheral embolization, and constitutional symptoms such as persistent low-grade fever, weakness, dizziness and anorexia, complicated in some cases by tumor infection.

Up to 20% of patients present with cardiac arrhythmias, most commonly atrial flutter or fibrillation, as seen in our case^2,3,13^. There is no pathognomonic finding from the physical examination. Given that myxomas can mimic numerous clinical entities, high clinical suspicion combined with cardiac imaging remains essential for diagnosis.

Echocardiography, especially transoesophageal echocardiography, remains the cornerstone of imaging for diagnosis, tumor localization and assessment of cardiac and valvular function. On contrast-enhanced computed tomography, cardiac myxomas typically appear as well-defined spherical masses with smooth or lobulated contours, heterogeneous contrast enhancement, and occasional calcifications^2–4^.

Contrast-enhanced cardiac MRI remains the most sensitive diagnostic modality for detecting cardiac masses, which typically demonstrate heterogeneous signal intensity (isointense or hyperintense lesions) reflecting the underlying tissue composition. Low signal intensity on post-contrast sequences may indicate necrotic regions, whereas areas of marked enhancement suggest increased vascularity and inflammation.

Histopathological examination following biopsy remains the gold standard for diagnosis. Cardiac myxomas characteristically demonstrate a gelatinous myxoid stroma rich in mucopolysaccharides, collagen type IV, and elastin. These lesions contain a heterogeneous population of cells, including stellate and spindle-shaped cells exhibiting features consistent with mesenchymal progenitors. Mitotic figures are rare, and cytologic atypia and necrosis are typically absent.

Degenerative changes observed in cardiac myxomas include hemorrhage, fibrosis, thrombosis, and calcification. Necrosis occurs rarely, with an incidence of less than 3% ^2,3^. Histologically, myxomas are typically covered by flattened endothelial cells and contain thin-walled blood vessels lacking pericytes.

Cardiac myxomas demonstrate increased expression of several biomarkers, including endothelin-1, interleukin-6 and -8 (IL-6, IL-8), C-X-C motif chemokine ligand 1 (CXCL1), EDN1, HAND1, MMP1, and VEGFR. This molecular profile provides support for the germ-line theory of myxoma pathogenesis.

Several theories have been proposed to explain the origin and pathogenesis of cardiac myxomas. The **neoplastic theory** accounts for the 5–10% of cases with a familial basis, often associated with Carney complex (CNC), an autosomal dominant syndrome caused by mutations in the *PRKAR1A* gene.

The **thrombotic theory** suggests that myxomas may originate from endothelial cells overlying organized thrombi, which undergo dysplastic transformation and acquire neoplastic characteristics.

The **metaplastic theory** proposes that myxomas arise through metaplasia of endocardial tissue, whereby one differentiated cell type transforms into another. This process may be triggered by mechanical stress, inflammation, or genetic mutations that induce a phenotypic switch, culminating in the characteristic myxoid tissue.

The **dysembryoplastic theory** posits that cardiac myxomas develop from embryonic remnants within the endocardium, specifically from undifferentiated mesenchymal cells retained from embryogenesis.

Finally, the **inflammatory theory** is supported by the overexpression of pro-inflammatory cytokines, particularly IL-6, in cardiac myxomas. A chronic inflammatory microenvironment may promote angiogenesis and activate cellular pathways that drive tumor formation and growth^2–4^.

### Diagnostic evaluation and imaging

Cardiac myxomas typically demonstrate growth rates of 1–4 mm per month, whereas growth exceeding five mm per month should raise suspicion for malignancy.

Additional clinical and imaging features suggestive of a malignant cardiac mass include: right atrial location, myocardial infiltration, coronary artery encasement, constitutional symptoms (particularly weight loss), pericardial effusion, severe valvular dysfunction, cardiac arrhythmias, superior vena cava obstruction, pulmonary embolism, and the presence of extracardiac masses^5–9^.

Differential diagnosis encompasses both benign and malignant cardiac masses. Benign lesions include mural thrombi and papillary fibroelastomas. Malignant primary cardiac tumors to consider include myxofibrosarcoma, angiosarcoma, and epithelioid haemangioendothelioma. Cardiac metastases, particularly from lymphoma, breast, or lung carcinoma, should also be excluded. Diagnostic complexity may arise when cardiac myxomas demonstrate atypical histological features, such as increased cellular atypia or hypercellularity, which can mimic malignant transformation and complicate tissue diagnosis^10–12^.

Multimodality imaging plays a crucial role in the noninvasive diagnosis and characterization of cardiac masses. Echocardiography remains the initial imaging modality for patients with suspected cardiac masses. Cardiac magnetic resonance (CMR) imaging is considered the gold standard for tissue characterization and comprehensive evaluation. Cardiac computed tomography (CT), either in combination with or as an alternative to CMR, provides critical information for specific clinical scenarios and presurgical planning. In case of inconclusive results, ^18^F-FDG PET is invaluable for assessing malignant potential and staging cardiac tumors.

In clinical practice, the diagnostic approach is often influenced by the availability of local resources and institutional expertise. Raising awareness of the benefits of multimodality imaging is therefore essential, particularly for complex cases. The selection of imaging modalities should be tailored to individual clinical scenarios, guided by the diagnostic question, institutional capabilities, and patient-specific factors^14,15^.

## Case summary

In our case, a 77-year-old male patient with a history of non-Hodgkin lymphoma treated with chemotherapy 15 years ago presented with recurrent episodes of symptomatic atrial fibrillation. The transthoracic echocardiography depicted a mass within the left atrium of maximum diameter around 25 mm which was absent 6 months ago. This growth rate of approximately five mm/month was not fully consistent with typical benign features of a myxoma. In the context of multimodality imaging, most of the features from the rest of the imaging techniques used were suggestive of a cardiac myxoma with atypical characteristics. Despite that, a lymphoma recurrence given the patients history and rapid growth rate of the mass, as well as the absence of a lymph node for a potential biopsy could not be excluded. A FDG-PET was not carried out due to limited availability. As a result, the decision surgical excursion of the mass was taken in the context of the Heart Team, with the participation of the patient and taking into consideration his concerns, as well as the symptomatic nature of the cardiac mass. Thoracoscopic resection of the tumor was carried out successfully, given the cardiac surgeons expertise and patient’s preference, and being totally in line with the modern approach of cardiac myxomas excision with minimally invasive/robotic techniques as an alternative to the open surgery and sternotomy for aesthetic and quality of life reasons^16,17^. Finally, biopsy revealed necrosis of the mass, which is a rare histopathological feature of CM, which may have been contributing to the rapid increase in the mass size.

## What have we learned?

Cardiac myxoma remains a complex and diverse clinical entity, mimicking many diseases, and so clinical suspicion is of outmost importance for its diagnosis. In the present case, the rapid growth rate of the myxoma—likely attributable to intratumoral necrosis, a rare occurrence in cardiac myxomas—initially suggested cardiac lymphoma recurrence in a patient with a history of non-Hodgkin lymphoma, illustrating the diagnostic complexity of these lesions.

The use of multimodality imaging is crucial in the diagnostic procedure of intracardiac masses, assisting the differential diagnosis and in many cases the characterization of the masses.

However, when diagnostic uncertainty persists, a personalized, multidisciplinary approach through Heart Team discussion, with patient involvement in shared decision-making, remains the cornerstone of optimal management for complex clinical entities such as cardiac myxomas^16,17^.

## Funding

No funding was received.

## Conflicts of interest/Competing interests

The authors declare that they have no conflict of interest.

## Informed consent

Patient’s informed consent for publication of the manuscript was obtained.

## Authorship contribution

Authors contributed equally to writing, reviewing, drafting, image editing and conceptualization of this manuscript.
